# Supplementary figures and images for: Deep conservation of prion-like composition in the eukaryotic prion-former Pub1/Tia1 family and its relatives
Source: PeerJ. 2020 Apr 17;8:e9023. doi: 10.7717/peerj.9023 (PMC7169965; doi:10.7717/peerj.9023)

Tree scale: 1

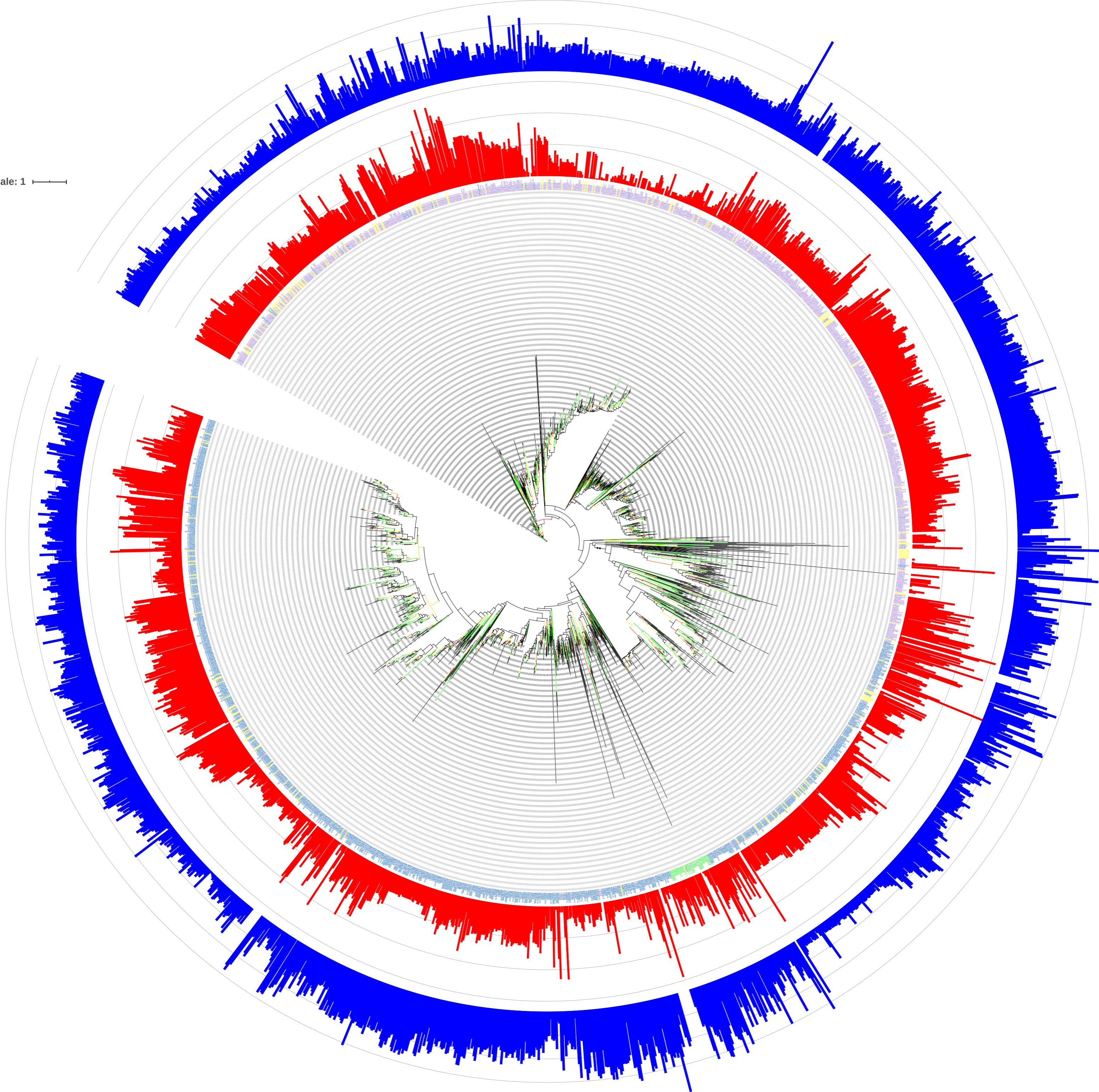

Supplement: Supplemental Information 3 — This is made using RaxML (Stamatakis, 2015), as described in “Methods”, and drawn using iTOL (Letunic & Bork, 2019). The bootstrap values are colour-coded with gradient colouring for 0.5–1.0, ranging from red for 0.5 to green for 1.0, with <0.5 black. The UniProt (UniProt, 2019) accession numbers for proteins are used if available, but otherwise the protein identifier from the OrthoDB database is used (Kriventseva et al., 2019). Pub1/Tia1/Tiar orthologs are coloured purple, Nam8 orthologs blue and Ngr1 green. The identifiers of additional paralogs are coloured yellow. The circles of annotations are as follows in order from inside to outside: (i) PLAAC (Lancaster et al., 2014) LLR prion-like composition score (red); (ii) percentage of residues annotated as disordered by IUPred2a (Dosztanyi, 2018) (blue). [file peerj-08-9023-s003.pdf]
