# Supplementary material for: Deep conservation of prion-like composition in the eukaryotic prion-former Pub1/Tia1 family and its relatives
Source: PeerJ. 2020 Apr 17;8:e9023. doi: 10.7717/peerj.9023 (PMC7169965; doi:10.7717/peerj.9023)
Supplement: Supplemental Information 4 — BLASTP output showing the most similar proteins to Pub1, Ngr1 and Nam8 in the yeast proteome. [file peerj-08-9023-s004.pdf]

Standard BLASTP output for comparing Pub1 to the yeast proteome (from the NCBI blast server https://blast.ncbi.nlm.nih.gov/Blast.cgi; the lines for Pab1 are highlighted in yellow)

Select for downloading or viewing reports	Description	Max Score	Total Score	Query Cover	E value	Per. Ident	Accession
Select seq ref|NP_014382.1|	Pub1p [Saccharomyces cerevisiae S288C]	931	931	100%	0.0	100.00%	NP_014382.1
Select seq ref|NP_011954.1|	Nam8p [Saccharomyces cerevisiae S288C]	108	108	75%	4e-26	29.65%	NP_011954.1
Select seq ref|NP_009771.3|	Ngr1p [Saccharomyces cerevisiae S288C]	97.1	97.1	66%	6e-22	27.67%	NP_009771.3
Select seq pdb|6R5K|D	Chain D, Polyadenylate-binding protein, cytoplasmic and nuclear [Saccharomyces cerevisiae S288C]	90.1	173	41%	1e-19	26.60%	6R5K_D
Select seq ref|NP_011092.1|	polyadenylate-binding protein [Saccharomyces cerevisiae S288C]	90.1	173	41%	1e-19	26.60%	NP_011092.1
Select seq ref|NP_011675.1|	Nsr1p [Saccharomyces cerevisiae S288C]	82.4	149	38%	2e-17	26.09%	NP_011675.1
Select seq ref|NP_014518.1|	Hrp1p [Saccharomyces cerevisiae S288C]	65.5	110	36%	6e-12	26.04%	NP_014518.1
Select seq ref|NP_116678.1|	Pes4p [Saccharomyces cerevisiae S288C]	58.5	58.5	20%	1e-09	34.74%	NP_116678.1
Select seq ref|NP_011879.1|	Mip6p [Saccharomyces cerevisiae S288C]	55.1	55.1	24%	1e-08	30.28%	NP_011879.1

)

Standard BLASTP output for comparing Nam8 to the yeast proteome (as above)

Select seq ref|NP_011954.1|	Nam8p [Saccharomyces cerevisiae S288C]	1067	1067	100%	0.0	100.00%	NP_011954.1
Select seq ref|NP_009771.3|	Ngr1p [Saccharomyces cerevisiae S288C]	189	189	67%	2e-53	30.28%	NP_009771.3
Select seq ref|NP_014382.1|	Pub1p [Saccharomyces cerevisiae S288C]	78.6	78.6	35%	4e-16	34.03%	NP_014382.1
Select seq pdb|6R5K|D	Chain D, Polyadenylate-binding protein, cytoplasmic and nuclear [Saccharomyces cerevisiae S288C]	66.6	66.6	69%	4e-12	21.38%	6R5K_D
Select seq ref|NP_011092.1|	polyadenylate-binding protein [Saccharomyces cerevisiae S288C]	66.6	66.6	69%	5e-12	21.38%	NP_011092.1
Select seq ref|NP_014964.1|	U2 snRNP complex subunit HSH49 [Saccharomyces cerevisiae S288C]	53.5	84.3	63%	1e-08	23.81%	NP_014964.1
Select seq pdb|5LSB|A	Chain A, Protein Hsh49 [Saccharomyces cerevisiae S288C]	53.1	85.9	65%	2e-08	23.81%	5LSB_A
Select seq ref|NP_014518.1|	Hrp1p [Saccharomyces cerevisiae S288C]	46.2	130	24%	1e-05	38.89%	NP_014518.1
Select seq ref|NP_011471.1|	Rna15p [Saccharomyces cerevisiae S288C]	40.8	40.8	14%	4e-04	29.87%	NP_011471.1

Standard BLASTP output for comparing Ngr1 to the yeast proteome (as above)

Description	Max Score	Total Score	Query Cover	E value	Per. Ident	Accession
Select seq ref|NP_009771.3|	Ngr1p [Saccharomyces cerevisiae S288C]	1385	1385	100%	0.0	100.00%	NP_009771.3
Select seq ref|NP_011954.1|	Nam8p [Saccharomyces cerevisiae S288C]	194	194	62%	1e-54	31.15%	NP_011954.1
Select seq ref|NP_014382.1|	Pub1p [Saccharomyces cerevisiae S288C]	70.5	70.5	21%	3e-13	35.17%	NP_014382.1
Select seq ref|NP_011092.1|	polyadenylate-binding protein [Saccharomyces cerevisiae S288C]	55.8	55.8	13%	1e-08	33.33%	NP_011092.1
Select seq pdb|6R5K|D	Chain D, Polyadenylate-binding protein, cytoplasmic and nuclear [Saccharomyces cerevisiae S288C]	55.8	55.8	13%	2e-08	33.33%	6R5K_D
Select seq ref|NP_014518.1|	Hrp1p [Saccharomyces cerevisiae S288C]	44.7	44.7	11%	4e-05	30.49%	NP_014518.1
Select seq ref|NP_014148.1|	Nrd1 complex RNA-binding subunit [Saccharomyces cerevisiae S288C]	43.9	43.9	10%	7e-05	32.86%	NP_014148.1
Select seq ref|NP_011766.3|	Rie1p [Saccharomyces cerevisiae S288C]	40.0	40.0	10%	0.001	30.26%	NP_011766.3
Select seq ref|NP_009819.1|	Rgd1p [Saccharomyces cerevisiae S288C]	28.1	28.1	11%	7.0	28.57%	NP_009819.1
